# (*E*)-3-(4-Methyl­phen­yl)-3-[3-(4-methyl­phen­yl)-1*H*-pyrazol-1-yl]-2-propenal

**DOI:** 10.1107/S1600536808034697

**Published:** 2008-10-31

**Authors:** P. Ramesh, A. Subbiahpandi, Ramaiyan Manikannan, S. Muthusubramanian, M. N. Ponnuswamy

**Affiliations:** a Department of Physics, Presidency College (Autonomous), Chennai 600 005, India; b Department of Organic Chemistry, School of Chemistry, Madurai Kamaraj University, Madurai 625 021, India; c Centre of Advanced Study in Crystallography and Biophysics, University of Madras, Guindy Campus, Chennai 600 025, India

## Abstract

In the title compound, C_20_H_18_N_2_O, the pyrazole ring adopts a planar conformation. The C—N bond lengths in the pyrazole ring are shorter than a standard C—N single bond (1.443 Å), but longer than a standard double bond (1.269 Å), indicating electron delocalization. The propenal group assumes an extended conformation. Inter­molecular C—H⋯O hydrogen bonds connect mol­ecules into cyclic centrosymmetric *R*_2_^2^(26) dimers, which are cross-linked *via* C—H⋯π inter­actions.

## Related literature

For the properties of pyrazole derivatives, see: Baraldi *et al.* (1998 ▶); Bruno *et al.* (1990 ▶); Chen & Li (1998 ▶); Cottineau *et al.* (2002 ▶); Londershausen (1996 ▶); Mishra *et al.* (1998 ▶); Smith *et al.* (2001 ▶). For related literature, see: Beddoes *et al.* (1986 ▶); Jin *et al.* (2004 ▶); Bernstein *et al.* (1995 ▶); Cordell (1981 ▶).
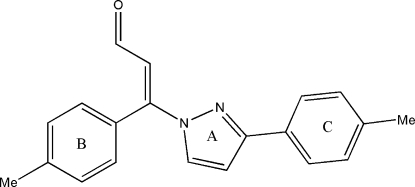


## Experimental

### 

#### Crystal data

C_20_H_18_N_2_O
*M* *_r_* = 302.36
Triclinic, 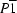

*a* = 10.0560 (9) Å
*b* = 10.0786 (8) Å
*c* = 10.3176 (9) Å
α = 62.040 (4)°
β = 79.356 (4)°
γ = 63.038 (4)°
*V* = 822.73 (12) Å^3^
*Z* = 2
Mo *K*α radiation
μ = 0.08 mm^−1^
*T* = 293 (2) K
0.30 × 0.22 × 0.20 mm

#### Data collection

Bruker APEXII CCD area-detector diffractometer
Absorption correction: multi-scan (*SADABS*; Sheldrick, 2001 ▶) *T*_min_ = 0.980, *T*_max_ = 0.985
14086 measured reflections
2887 independent reflections
2315 reflections with *I* > 2σ(*I*)
*R*_int_ = 0.035

#### Refinement

*R*[*F* ^2^ > 2σ(*F* ^2^)] = 0.048
*wR*(*F* ^2^) = 0.154
*S* = 1.03
2887 reflections
210 parameters
H-atom parameters constrained
Δρ_max_ = 0.24 e Å^−3^
Δρ_min_ = −0.23 e Å^−3^

### 

Data collection: *APEX2* (Bruker, 2004 ▶); cell refinement: *APEX2*; data reduction: *SAINT* (Bruker, 2004 ▶); program(s) used to solve structure: *SHELXS97* (Sheldrick, 2008 ▶); program(s) used to refine structure: *SHELXL97* (Sheldrick, 2008 ▶); molecular graphics: *ORTEP-3* (Farrugia, 1997 ▶); software used to prepare material for publication: *SHELXL97* and *PLATON* (Spek, 2003 ▶).

## Supplementary Material

Crystal structure: contains datablocks global, I. DOI: 10.1107/S1600536808034697/gw2051sup1.cif

Structure factors: contains datablocks I. DOI: 10.1107/S1600536808034697/gw2051Isup2.hkl

Additional supplementary materials:  crystallographic information; 3D view; checkCIF report

## Figures and Tables

**Table 1:** Hydrogen-bond geometry (Å, °)

*D*—H⋯*A*	*D*—H	H⋯*A*	*D*⋯*A*	*D*—H⋯*A*
C22—H22*B*⋯O1^i^	0.96	2.60	3.446 (3)	148
C9—H9⋯*Cg*1^ii^	0.93	2.80	3.690 (3)	161

Symmetry codes: (i) 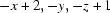; (ii) 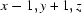. *Cg*1 is the centroid of the C16–C21 ring.

**Fig. 1.**
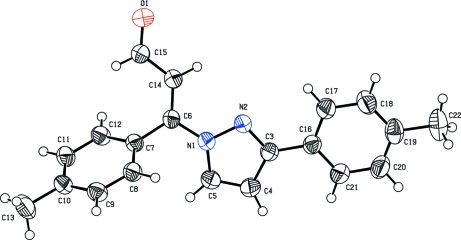
Perspective view of the molecule showing the thermal ellipsoids are drawn at 50% probability level. The H atoms are shown as small circles of arbitrary radii.

**Fig. 2.**
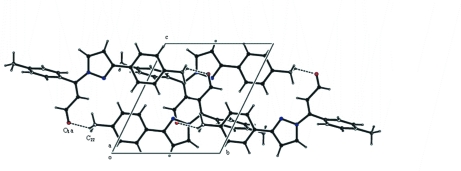
The crystal packing of the molecules viewed down the a axis.

## supplementary crystallographic information

### Comment

Some pyrazole derivatives are successfully tested for their antifungal (Chen & Li, 1998), antihistaminic (Mishra *et al.*, 1998) and anti-inflammatory (Smith *et al.*, 2001) properties. These derivatives also possess significant antiarrhythmic and sedative (Bruno *et al.*, 1990), hypoglycemic (Cottineau *et al.*, 2002), antiviral (Baraldi *et al.*, 1998), and pesticidal (Londershausen,1996) activities.

The pyrazole ring adopts planar conformation. The sum of the angles at N1 of the pyrazole ring (360.0°) is in accordance with *sp*^2^ hybridization (Beddoes *et al.*, 1986). The C—N bond lengths in the pyrazole ring are 1.321 (2) and 1.360 (2) Å, which are shorter than a C—N single bond length of 1.443 Å, but longer than a double bond length of 1.269 Å, (Jin *et al.*, 2004), indicating electron delocalization. The pyrazole ring A and methylphenyl ring C are near-coplanar with the inter-ring dihedral angle of 4.50 (13)°, whereas the pyrazole ring is twisted by an angle of 66.31 (12)° to the methylphenyl ring B. The propenal group assumes an extended conformation which is evidenced from the torsion angles [N1—C6—C14—C15]-169.74 (16) ° and [C5—N1—C6—C14]-160.85 (19)°. The crystal packing is stabilized by C—H···O and C—H-π interactions in addition to van der Waals forces. The molecules at (*x*, *y*, *z*) and (2 - *x*,-*y*,1 - *z*) are linked by C22—H22B···O1 hydrogen bonds into cyclic cenrosymmetric *R*_2_^2^(26) dimers,

### Experimental

The mixture of 1-(4-methylphenyl)-1-ethanone *N*-[(*E*)-1-phenylethylidene] hydrazone (0.003 mole) and 3 ml of dimethyl formamide kept in an ice bath at 0° C, phosphorus oxycholride (0.024 mole) was added dropwise for 5–10 minutes. The reaction mixture was then kept in a microwave oven at 600 W for 30–60 sec. The process of the reaction was monitored by TLC. After completion of the reaction, the reaction mixture was poured into crushed ice and extracted with dichloromethane. The organic layer was dried with anhydrous sodium sulfate. The different compounds present in the mixture were separated by column chromatography using petroleum ether and ethyl acetate mixture as eluent. This isolated compound was recrystallized in dichloromethane to obtain (*E*)-3-(4-methylphenyl)-3-[3-(4-methylphenyl)-1H -pyrazol-1-yl]-2-propenal in 34% yield.

### Refinement

H atoms were positioned geometrically (C—H=0.93–0.96 Å)and allowed to ride on their parent atoms, with *U*_iso_(H) = 1.5*U*_eq_(C) for methyl H, 1.2*U*_eq_(C) for other H atoms.

### Crystal data

**Table:**

C_20_H_18_N_2_O	*Z* = 2
*M**_r_* = 302.36	*F*(000) = 320
Triclinic, *P*1	*D*_x_ = 1.221 Mg m^−^^3^
Hall symbol: -P 1	Mo *K*α radiation, λ = 0.71073 Å
*a* = 10.0560 (9) Å	Cell parameters from 2865 reflections
*b* = 10.0786 (8) Å	θ = 2.2–25.0°
*c* = 10.3176 (9) Å	µ = 0.08 mm^−^^1^
α = 62.040 (4)°	*T* = 293 K
β = 79.356 (4)°	Block, colorless
γ = 63.038 (4)°	0.30 × 0.22 × 0.20 mm
*V* = 822.73 (12) Å^3^	

### Data collection

**Table:**

Bruker APEXII CCD area-detector diffractometer	2887 independent reflections
Radiation source: fine-focus sealed tube	2315 reflections with *I* > 2σ(*I*)
graphite	*R*_int_ = 0.035
ω and φ scans	θ_max_ = 25.0°, θ_min_ = 2.2°
Absorption correction: multi-scan (SADABS; Sheldrick, 2001)	*h* = −11→11
*T*_min_ = 0.980, *T*_max_ = 0.985	*k* = −11→11
14086 measured reflections	*l* = −12→12

### Refinement

**Table:**

Refinement on *F*^2^	Primary atom site location: structure-invariant direct methods
Least-squares matrix: full	Secondary atom site location: difference Fourier map
*R*[*F*^2^ > 2σ(*F*^2^)] = 0.048	Hydrogen site location: inferred from neighbouring sites
*wR*(*F*^2^) = 0.154	H-atom parameters constrained
*S* = 1.03	*w* = 1/[σ^2^(*F*_o_^2^) + (0.0885*P*)^2^ + 0.2614*P*] where *P* = (*F*_o_^2^ + 2*F*_c_^2^)/3
2887 reflections	(Δ/σ)_max_ = 0.037
210 parameters	Δρ_max_ = 0.24 e Å^−^^3^
0 restraints	Δρ_min_ = −0.23 e Å^−^^3^

### Special details

**Table:**

Geometry. All e.s.d.'s (except the e.s.d. in the dihedral angle between two l.s. planes) are estimated using the full covariance matrix. The cell e.s.d.'s are taken into account individually in the estimation of e.s.d.'s in distances, angles and torsion angles; correlations between e.s.d.'s in cell parameters are only used when they are defined by crystal symmetry. An approximate (isotropic) treatment of cell e.s.d.'s is used for estimating e.s.d.'s involving l.s. planes.
Refinement. Refinement of *F*^2^ against ALL reflections. The weighted *R*-factor *wR* and goodness of fit *S* are based on *F*^2^, conventional *R*-factors *R* are based on *F*, with *F* set to zero for negative *F*^2^. The threshold expression of *F*^2^ > σ(*F*^2^) is used only for calculating *R*-factors(gt) *etc*. and is not relevant to the choice of reflections for refinement. *R*-factors based on *F*^2^ are statistically about twice as large as those based on *F*, and *R*- factors based on ALL data will be even larger.

### Fractional atomic coordinates and isotropic or equivalent isotropic displacement parameters (Å^2^)

**Table:**

	*x*	*y*	*z*	*U*_iso_*/*U*_eq_	
O1	0.67828 (17)	0.53579 (18)	0.72181 (16)	0.0625 (4)	
N1	0.60777 (16)	0.58095 (17)	0.26253 (16)	0.0432 (4)	
N2	0.68095 (16)	0.41223 (17)	0.31521 (16)	0.0427 (4)	
C3	0.69279 (19)	0.3841 (2)	0.19979 (19)	0.0416 (4)	
C4	0.6304 (2)	0.5334 (2)	0.0717 (2)	0.0519 (5)	
H4	0.6267	0.5457	−0.0229	0.062*	
C5	0.5774 (2)	0.6543 (2)	0.1158 (2)	0.0515 (5)	
H5	0.5288	0.7675	0.0565	0.062*	
C6	0.57725 (18)	0.6542 (2)	0.35731 (19)	0.0400 (4)	
C7	0.45725 (19)	0.8246 (2)	0.30619 (18)	0.0396 (4)	
C8	0.3162 (2)	0.8596 (2)	0.2667 (2)	0.0450 (4)	
H8	0.2986	0.7771	0.2657	0.054*	
C9	0.2021 (2)	1.0156 (2)	0.2292 (2)	0.0477 (5)	
H9	0.1077	1.0364	0.2051	0.057*	
C10	0.2255 (2)	1.1425 (2)	0.2264 (2)	0.0472 (5)	
C11	0.3665 (2)	1.1077 (2)	0.2624 (2)	0.0490 (5)	
H11	0.3847	1.1916	0.2597	0.059*	
C12	0.4818 (2)	0.9513 (2)	0.3023 (2)	0.0451 (4)	
H12	0.5759	0.9308	0.3267	0.054*	
C13	0.1000 (3)	1.3122 (3)	0.1849 (3)	0.0705 (6)	
H13A	0.1319	1.3799	0.2011	0.106*	
H13B	0.0158	1.3034	0.2439	0.106*	
H13C	0.0719	1.3618	0.0831	0.106*	
C14	0.65218 (19)	0.5672 (2)	0.48724 (19)	0.0439 (4)	
H14	0.7338	0.4666	0.5039	0.053*	
C15	0.6144 (2)	0.6193 (2)	0.6020 (2)	0.0465 (4)	
H15	0.5367	0.7228	0.5837	0.056*	
C16	0.75914 (19)	0.2135 (2)	0.21612 (19)	0.0431 (4)	
C17	0.8059 (2)	0.0785 (2)	0.3526 (2)	0.0541 (5)	
H17	0.7965	0.0953	0.4357	0.065*	
C18	0.8664 (2)	−0.0813 (3)	0.3660 (2)	0.0600 (5)	
H18	0.8978	−0.1702	0.4583	0.072*	
C19	0.8812 (2)	−0.1120 (3)	0.2463 (3)	0.0563 (5)	
C20	0.8340 (2)	0.0235 (3)	0.1112 (3)	0.0614 (6)	
H20	0.8430	0.0064	0.0284	0.074*	
C21	0.7740 (2)	0.1837 (3)	0.0956 (2)	0.0548 (5)	
H21	0.7432	0.2722	0.0029	0.066*	
C22	0.9457 (3)	−0.2851 (3)	0.2607 (3)	0.0817 (8)	
H22A	0.9448	−0.3606	0.3615	0.123*	
H22B	1.0465	−0.3148	0.2285	0.123*	
H22C	0.8869	−0.2905	0.2013	0.123*	

### Atomic displacement parameters (Å^2^)

**Table:**

	*U*^11^	*U*^22^	*U*^33^	*U*^12^	*U*^13^	*U*^23^
O1	0.0725 (10)	0.0569 (9)	0.0525 (9)	−0.0170 (7)	−0.0167 (7)	−0.0231 (7)
N1	0.0508 (9)	0.0338 (8)	0.0399 (8)	−0.0144 (6)	−0.0021 (6)	−0.0144 (6)
N2	0.0462 (8)	0.0361 (8)	0.0409 (8)	−0.0128 (6)	−0.0014 (6)	−0.0167 (6)
C3	0.0412 (9)	0.0424 (10)	0.0394 (9)	−0.0158 (8)	0.0026 (7)	−0.0187 (8)
C4	0.0643 (12)	0.0491 (11)	0.0379 (10)	−0.0213 (9)	0.0008 (8)	−0.0182 (8)
C5	0.0641 (12)	0.0397 (10)	0.0405 (10)	−0.0178 (9)	−0.0035 (8)	−0.0119 (8)
C6	0.0423 (9)	0.0363 (9)	0.0435 (9)	−0.0187 (8)	0.0016 (7)	−0.0173 (7)
C7	0.0441 (9)	0.0334 (9)	0.0386 (9)	−0.0171 (7)	−0.0002 (7)	−0.0127 (7)
C8	0.0505 (10)	0.0400 (10)	0.0487 (10)	−0.0225 (8)	−0.0043 (8)	−0.0172 (8)
C9	0.0428 (10)	0.0464 (10)	0.0493 (10)	−0.0182 (8)	−0.0047 (8)	−0.0161 (8)
C10	0.0526 (11)	0.0360 (9)	0.0432 (10)	−0.0146 (8)	−0.0030 (8)	−0.0121 (8)
C11	0.0620 (12)	0.0333 (9)	0.0515 (11)	−0.0230 (9)	−0.0056 (9)	−0.0131 (8)
C12	0.0458 (10)	0.0401 (10)	0.0494 (10)	−0.0220 (8)	−0.0037 (8)	−0.0140 (8)
C13	0.0647 (14)	0.0433 (12)	0.0827 (16)	−0.0084 (10)	−0.0120 (11)	−0.0206 (11)
C14	0.0445 (10)	0.0376 (9)	0.0472 (10)	−0.0147 (8)	−0.0026 (8)	−0.0179 (8)
C15	0.0504 (10)	0.0400 (10)	0.0494 (11)	−0.0177 (8)	−0.0057 (8)	−0.0185 (8)
C16	0.0397 (9)	0.0449 (10)	0.0449 (10)	−0.0164 (8)	0.0043 (7)	−0.0224 (8)
C17	0.0643 (12)	0.0479 (11)	0.0458 (11)	−0.0184 (9)	0.0031 (9)	−0.0231 (9)
C18	0.0643 (13)	0.0425 (11)	0.0598 (13)	−0.0175 (10)	0.0041 (10)	−0.0179 (9)
C19	0.0412 (10)	0.0528 (12)	0.0819 (15)	−0.0178 (9)	0.0089 (9)	−0.0391 (11)
C20	0.0588 (12)	0.0667 (14)	0.0698 (14)	−0.0182 (11)	0.0022 (10)	−0.0463 (12)
C21	0.0574 (12)	0.0539 (12)	0.0506 (11)	−0.0145 (9)	−0.0029 (9)	−0.0280 (9)
C22	0.0724 (16)	0.0617 (15)	0.123 (2)	−0.0251 (12)	0.0162 (15)	−0.0569 (16)

### Geometric parameters (Å, °)

**Table:**

O1—C15	1.215 (2)	C12—H12	0.9300
N1—C5	1.360 (2)	C13—H13A	0.9600
N1—N2	1.369 (2)	C13—H13B	0.9600
N1—C6	1.399 (2)	C13—H13C	0.9600
N2—C3	1.321 (2)	C14—C15	1.435 (3)
C3—C4	1.411 (3)	C14—H14	0.9300
C3—C16	1.469 (2)	C15—H15	0.9300
C4—C5	1.348 (3)	C16—C21	1.379 (3)
C4—H4	0.9300	C16—C17	1.387 (3)
C5—H5	0.9300	C17—C18	1.384 (3)
C6—C14	1.344 (2)	C17—H17	0.9300
C6—C7	1.480 (2)	C18—C19	1.375 (3)
C7—C12	1.388 (2)	C18—H18	0.9300
C7—C8	1.388 (2)	C19—C20	1.380 (3)
C8—C9	1.377 (3)	C19—C22	1.501 (3)
C8—H8	0.9300	C20—C21	1.380 (3)
C9—C10	1.388 (3)	C20—H20	0.9300
C9—H9	0.9300	C21—H21	0.9300
C10—C11	1.376 (3)	C22—H22A	0.9600
C10—C13	1.503 (3)	C22—H22B	0.9600
C11—C12	1.382 (3)	C22—H22C	0.9600
C11—H11	0.9300		
			
C5—N1—N2	110.87 (14)	C10—C13—H13B	109.5
C5—N1—C6	129.15 (15)	H13A—C13—H13B	109.5
N2—N1—C6	119.97 (14)	C10—C13—H13C	109.5
C3—N2—N1	104.85 (14)	H13A—C13—H13C	109.5
N2—C3—C4	111.37 (16)	H13B—C13—H13C	109.5
N2—C3—C16	120.39 (16)	C6—C14—C15	124.24 (16)
C4—C3—C16	128.20 (16)	C6—C14—H14	117.9
C5—C4—C3	105.21 (17)	C15—C14—H14	117.9
C5—C4—H4	127.4	O1—C15—C14	123.68 (17)
C3—C4—H4	127.4	O1—C15—H15	118.2
C4—C5—N1	107.70 (16)	C14—C15—H15	118.2
C4—C5—H5	126.2	C21—C16—C17	118.12 (17)
N1—C5—H5	126.2	C21—C16—C3	120.56 (17)
C14—C6—N1	119.58 (15)	C17—C16—C3	121.31 (16)
C14—C6—C7	124.72 (15)	C18—C17—C16	120.45 (19)
N1—C6—C7	115.65 (14)	C18—C17—H17	119.8
C12—C7—C8	118.61 (16)	C16—C17—H17	119.8
C12—C7—C6	120.61 (15)	C19—C18—C17	121.7 (2)
C8—C7—C6	120.73 (15)	C19—C18—H18	119.1
C9—C8—C7	120.48 (16)	C17—C18—H18	119.1
C9—C8—H8	119.8	C18—C19—C20	117.22 (19)
C7—C8—H8	119.8	C18—C19—C22	121.8 (2)
C8—C9—C10	121.20 (17)	C20—C19—C22	120.9 (2)
C8—C9—H9	119.4	C19—C20—C21	121.90 (19)
C10—C9—H9	119.4	C19—C20—H20	119.1
C11—C10—C9	117.95 (17)	C21—C20—H20	119.1
C11—C10—C13	121.45 (18)	C16—C21—C20	120.6 (2)
C9—C10—C13	120.60 (18)	C16—C21—H21	119.7
C10—C11—C12	121.59 (17)	C20—C21—H21	119.7
C10—C11—H11	119.2	C19—C22—H22A	109.5
C12—C11—H11	119.2	C19—C22—H22B	109.5
C11—C12—C7	120.15 (16)	H22A—C22—H22B	109.5
C11—C12—H12	119.9	C19—C22—H22C	109.5
C7—C12—H12	119.9	H22A—C22—H22C	109.5
C10—C13—H13A	109.5	H22B—C22—H22C	109.5
			
C5—N1—N2—C3	−0.79 (19)	C9—C10—C11—C12	−1.0 (3)
C6—N1—N2—C3	−179.57 (15)	C13—C10—C11—C12	179.22 (19)
N1—N2—C3—C4	1.1 (2)	C10—C11—C12—C7	0.4 (3)
N1—N2—C3—C16	−176.65 (15)	C8—C7—C12—C11	1.1 (3)
N2—C3—C4—C5	−1.1 (2)	C6—C7—C12—C11	−176.32 (16)
C16—C3—C4—C5	176.50 (18)	N1—C6—C14—C15	−169.74 (16)
C3—C4—C5—N1	0.5 (2)	C7—C6—C14—C15	7.8 (3)
N2—N1—C5—C4	0.1 (2)	C6—C14—C15—O1	176.16 (18)
C6—N1—C5—C4	178.78 (17)	N2—C3—C16—C21	−178.92 (17)
C5—N1—C6—C14	−160.85 (19)	C4—C3—C16—C21	3.7 (3)
N2—N1—C6—C14	17.7 (2)	N2—C3—C16—C17	2.4 (3)
C5—N1—C6—C7	21.4 (3)	C4—C3—C16—C17	−175.02 (19)
N2—N1—C6—C7	−160.09 (14)	C21—C16—C17—C18	0.4 (3)
C14—C6—C7—C12	54.5 (2)	C3—C16—C17—C18	179.17 (18)
N1—C6—C7—C12	−127.83 (17)	C16—C17—C18—C19	−0.5 (3)
C14—C6—C7—C8	−122.9 (2)	C17—C18—C19—C20	0.4 (3)
N1—C6—C7—C8	54.8 (2)	C17—C18—C19—C22	−179.5 (2)
C12—C7—C8—C9	−2.0 (3)	C18—C19—C20—C21	−0.1 (3)
C6—C7—C8—C9	175.42 (16)	C22—C19—C20—C21	179.7 (2)
C7—C8—C9—C10	1.4 (3)	C17—C16—C21—C20	−0.2 (3)
C8—C9—C10—C11	0.1 (3)	C3—C16—C21—C20	−178.93 (17)
C8—C9—C10—C13	179.87 (19)	C19—C20—C21—C16	0.0 (3)

### Hydrogen-bond geometry (Å, °)

**Table:**

*D*—H···*A*	*D*—H	H···*A*	*D*···*A*	*D*—H···*A*
C22—H22B···O1^i^	0.96	2.60	3.446 (3)	148
C9—H9···Cg1^ii^	0.93	2.80	3.690 (3)	161

Symmetry codes: (i) −*x*+2, −*y*, −*z*+1; (ii) *x*−1, *y*+1, *z*.

## References

[bb1] Baraldi, P. G., Manfredini, S., Romagnoli, R., Stevanato, L., Zaid, A. N. & Manservigi, R. (1998). *Nucleosides Nucleotides*, **17**, 2165–2171.

[bb2] Beddoes, R. L., Dalton, L., Joule, T. A., Mills, O. S., Street, J. D. & Watt, C. I. F. (1986). *J. Chem. Soc. Perkin Trans. 2*, pp. 787–797.

[bb3] Bernstein, J., Davis, R. E., Shimoni, L. & Chang, N. L. (1995). *Angew. Chem. Int. Ed. Engl.* **34**, 1555–1573.

[bb4] Bruker (2004). *APEX2* and *SAINT* Bruker AXS Inc., Madison, Wisconsin, USA.

[bb5] Bruno, O., Bondavalli, F., Ranise, A., Schenone, P., Losasso, C., Cilenti, L., Matera, C. & Marmo, E. (1990). *Farmaco*, **45**, 147–66. 2133992

[bb6] Chen, H. S. & Li, Z. M. (1998). *Chem. J. Chin. Univ.* **19**, 572–576.

[bb7] Cordell, G. (1981). *Introduction to Alkaloids: A Biogenic Approach* New York: Wiley International.

[bb8] Cottineau, B., Toto, P., Marot, C., Pipaud, A. & Chenault, J. (2002). *Bioorg. Med. Chem.* **12**, 2105–2108. 10.1016/s0960-894x(02)00380-3 12127514

[bb9] Farrugia, L. J. (1997). *J. Appl. Cryst.* **30**, 565.

[bb10] Jin, Z.-M., Li, L., Li, M.-C., Hu, M.-L. & Shen, L. (2004). *Acta Cryst.* C**60**, o642–o643. 10.1107/S0108270104016130 15345843

[bb11] Londershausen, M. (1996). *Pestic. Sci.* **48**, 269–274.

[bb12] Mishra, P. D., Wahidullah, S. & Kamat, S. Y. (1998). *Indian J. Chem. Sect. B*, **37**, 199.

[bb13] Sheldrick, G. M. (2001). *SADABS* University of Göttingen, Germany.

[bb14] Sheldrick, G. M. (2008). *Acta Cryst.* A**64**, 112–122. 10.1107/S0108767307043930 18156677

[bb15] Smith, S. R., Denhardt, G. & Terminelli, C. (2001). *Eur. J. Pharmacol.* **432**, 107–119. 10.1016/s0014-2999(01)01477-7 11734194

[bb16] Spek, A. L. (2003). *J. Appl. Cryst.* **36**, 7–13.

